# Insight into the role of 3D reconstruction of MRI imaging for complex anogenital hidradenitis suppurativa: A single-center case series

**DOI:** 10.1016/j.jpra.2026.03.011

**Published:** 2026-03-11

**Authors:** Amy Yoon, Danial Bahudin, Jeremy Pham, Thomas Lloyd, Erin Mcmeniman

**Affiliations:** a Division of Surgery, Princess Alexandra Hospital, Metro South Health, 199 Ipswich Road, Woolloongaba, QLD, Australia; b School of Medicine, Griffith University, 1 Parklands Drive, Southport, Gold Coast, Australia; c The University of Queensland, School of Medicine, Herston QLD 4006, Australia; d Department of Medical Imaging, Princess Alexandra Hospital, 199 Ipswich Road, Woolloongabba, QLD, Australia; e Dermatology, Princess Alexandra Hospital, 199 Ipswich Road, Woolloongabba, QLD, Australia

**Keywords:** Hidradenitis suppurativa, 3D modeling, Magnetic resonance imaging, Virtual surgical planning

## Abstract

**Background:**

Hidradenitis Suppurativa (HS) is a debilitating auto-inflammatory condition which typically occurs in the skin folds of the body such as the axilla, groin, gluteal and perianal regions. It is characterized by painful nodules which can develop fistulas and abscesses. Disease affecting the perianal region in particular requires multi-disciplinary input due to involvement of deeper structures. The HS multidisciplinary team (MDT) meetings held at Princess Alexandra Hospital (PAH) are held with the collaboration of dermatology, plastic surgery, colorectal surgery and radiology teams to manage complex HS cases across Queensland. Magnetic resonance imaging (MRI) is the modality of choice at this unit for patients with complex anogenital disease. An additional unique service that is offered at PAH is three-dimensional (3D) virtual modeling using these MRI scans. We believe that this provides a valuable tool for the MDT team in decision making and also for patients to better understand their disease. We aim to present a case series of eight patients with complex anogenital HS who received 3D virtual modeling of their MRI scans and outline our workflow for referrals.

**Discussion:**

We believe that 3D virtual reconstructions of MRI scans for complex anogenital HS cases are valuable in assisting decision making for clinicians. We hope that with ongoing optimizations in MRI sequences and advancements in 3D technology, this can become a more readily available tool for clinicians involved in the treatment of HS.

## Introduction

Hidradenitis suppurativa (HS) is a chronic auto-inflammatory disorder characterized by painful nodules which can progress to fistulas and abscesses that typically occur in skin folds of the body such as the axilla, groin, gluteal and perianal regions.^1^^,^^2^ It is a debilitating condition which affected approximately 0.67% of the adult Australian population in 2015.^3^ Genetic, environmental and behavioral factors are thought to influence the development of the disease. In total, 33% to 40% of individuals report an affected first-degree relative with many patients also reporting a history of autoimmune diseases such as Crohn’s disease, which is suggestive of a hereditary component.^4^ Environmental and behavioral influences include smoking, obesity, metabolic syndrome, and hormonal imbalances.

HS management involves a tailored approach of topical agents, oral antibiotics, immune modulators and, in the most severe cases, surgery. Severe cases require multidisciplinary team (MDT) input to optimize management. The HS MDT held at Princess Alexandra Hospital (PAH) is a unique service within the state of Queensland. Therefore, the majority of complex HS cases across Queensland are referred to this unit.

It can be challenging to clinically assess severity of tunnels and fistulous tracts and their depth of involvement.^5^ This often leads to an underestimate of the severity and depth of the disease. This is where imaging becomes extremely valuable in assisting decision making and risk assessment. There are two main modalities used in practice, ultrasound (US) and magnetic resonance imaging (MRI).

Ultrasound is readily accessible, safe and doesn’t expose individuals to radiation. The literature is very supportive of its use and its advantage in being able to detect subclinical features and superficial disease.^6^ The availability of the color Doppler imaging function also allows assessment of fistulous tracts.^7^ However, US imaging is very operator dependent and has depth limitations, reducing its ability to reliably assess deeper structures. Contact with the US probe can also cause pain.^7^

MRI uses a series of radiofrequency pulses within a static magnetic field to generate images without ionizing radiation. MRI is, however, expensive and time consuming and its diagnostic accuracy is heavily dependent on optimized imaging protocols. There are additionally certain contraindications for MRI such as implantable medical devices and claustrophobia. MRI’s role in superficial disease is limited.^7^ According to the literature, the role of MRI in HS is limited to assessment of severe cases of HS involving deeper tissues.^7^ In particular, it is beneficial for accurate characterization of perianal HS cases with potential involvement of the anal canal which are unable to be detected by US^6^ and require specialized surgical involvement.

Anogenital HS disease can occur in up to 32% of HS cases.^8^ Areas of particular importance in operative planning of anogenital disease are to determine whether there is anal sphincter complex, puborectalis, or presacral involvement. MRI offers superior visualization of these deeper structures. Kelly and Cronin^9^ describe the MRI findings of HS as relatively non-specific but that HS should be considered in the presence of sinus tracts, fistulas and/or scarring.^9^ We have found that the use of MRI is better served in the characterization of the extent of known HS rather than the initial diagnosis. With increasing understanding of the disease and surgical treatment, we have optimized the imaging protocol at our institution to allow visualization of the extent of HS in the pelvic area. This was done by utilizing a combination of routine perianal fistula imaging and thin slice volumetric acquisitions which allow multiplanar reconstruction without loss of detail. This facilitates multidisciplinary consideration of these complex cases which typically involve Dermatology, Radiology, Plastic and Colorectal surgical specialties.

In addition to the optimization of MRI sequences, we have also integrated 3D virtual modeling for complex HS cases at PAH. Whilst 3D modeling technology is becoming more prevalent in the medical field, its use is relatively novel for this indication.

Our group has published on the use of 3D virtual modeling and surgical navigation in the management of a case of severe buttock HS complicated by extensive deep SCC of the tracts.^10^ Almeida et al.^11^ have recently published on imaging of HS and described the use of 3D modeling in visualization of extent of disease. However, beyond this, the depiction of 3D modeling and advanced visualization of HS is limited in the literature.

In this report, we present our HS MDT format at PAH, the process by which referrals for 3D models are made and the workflow of creating these models. We then present a case series of seven patients in chronological order since 2023 who had 3D virtual reconstructions of their MRI scans which enhanced surgical planning and treatment decision making.

## Materials and methods

Ethical and site-specific approval was obtained from the Metro South Health with the reference number HREC/2025/QMS/122018. Verbal consent was obtained from all patients in accordance with the ethical application and study protocol.

**Figure 1:**
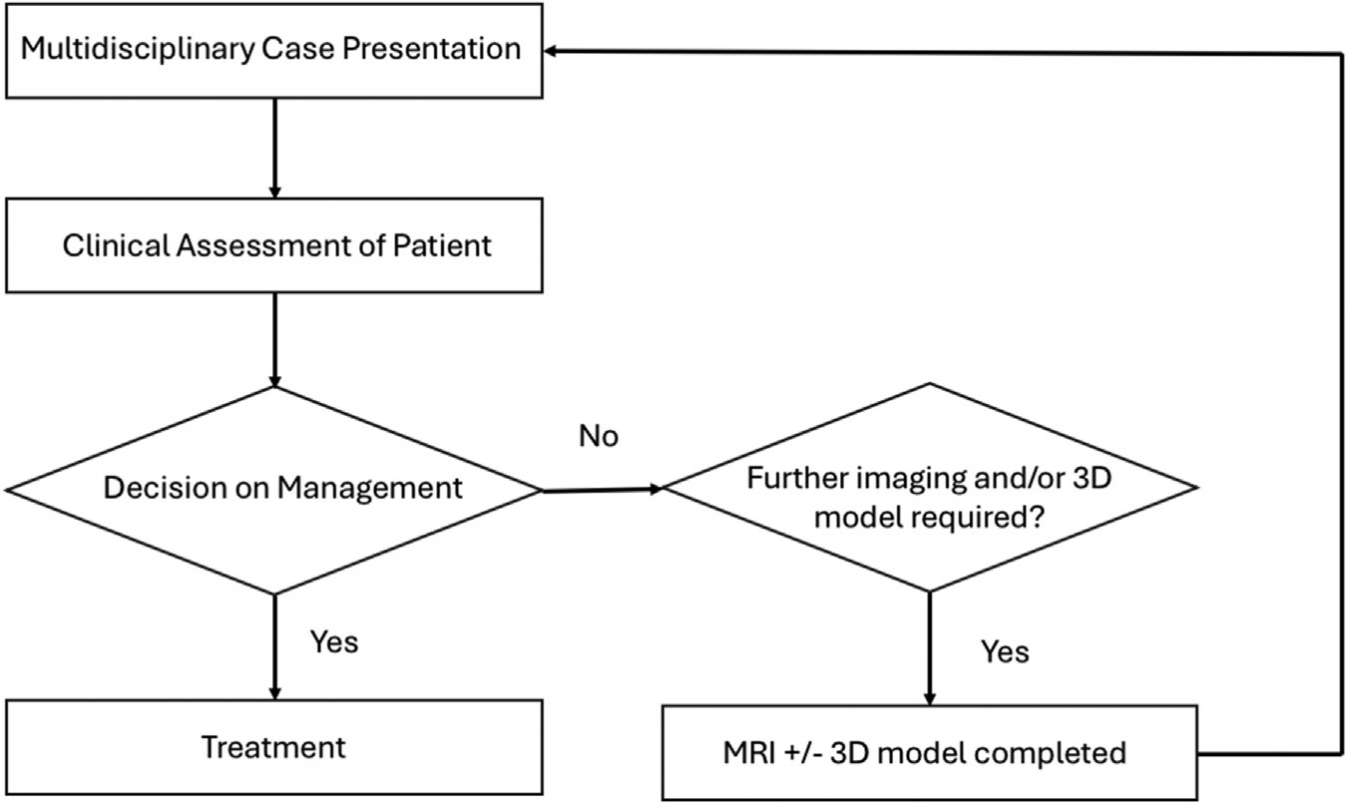
MDT and Referral process.

Our institution runs an HS MDT on a monthly basis. As the name suggests, it involves close collaboration between multiple specialties including dermatology, plastic surgery, colorectal surgery and radiology teams. Complex cases requiring multiple specialty input are identified by the dermatology team for discussion at this MDT. The discussion of each patient involves a summary of the patient’s history, review of clinical images, review of investigations and structural imaging if completed, discussion of treatment options followed by discussion with the patient about the outcome of the MDT discussion (Figure 1).

At the step where the radiologist presents structural imaging findings, any of the specialty teams may request a 3D virtual model of the MRI scan to help better visualize the patient’s anatomy and disease, particularly when considering surgical intervention in regions of complex or important anatomy. This includes the anal sphincteric complex or areas in close proximity to critical neurovascular structures.

The patient receives an MRI scan using our specific HS protocol. For those who live far away and prefer to have their imaging closer to home, a referral to an imaging center more locally can be made with instructions to contact the referring hospital for the protocol. The HS MRI protocol involves a high field strength MRI scanner (3 Tesla), and dedicated surface coils which facilitates increased image resolution for the assessment of skin disease. This in combination with thin slice acquisitions allow multiplanar reconstruction and accurate assessment of deeper structures as well.^9^ The sequences used for subsequent segmentation and delineation of extent of disease comprises thin slice T2 without fat saturation and thin slice post contrast fat saturated T1 weighted imaging. Both of these sequences are acquired with isometric voxels to allow multiplanar reformats without loss of image quality.

**Figure 2:**
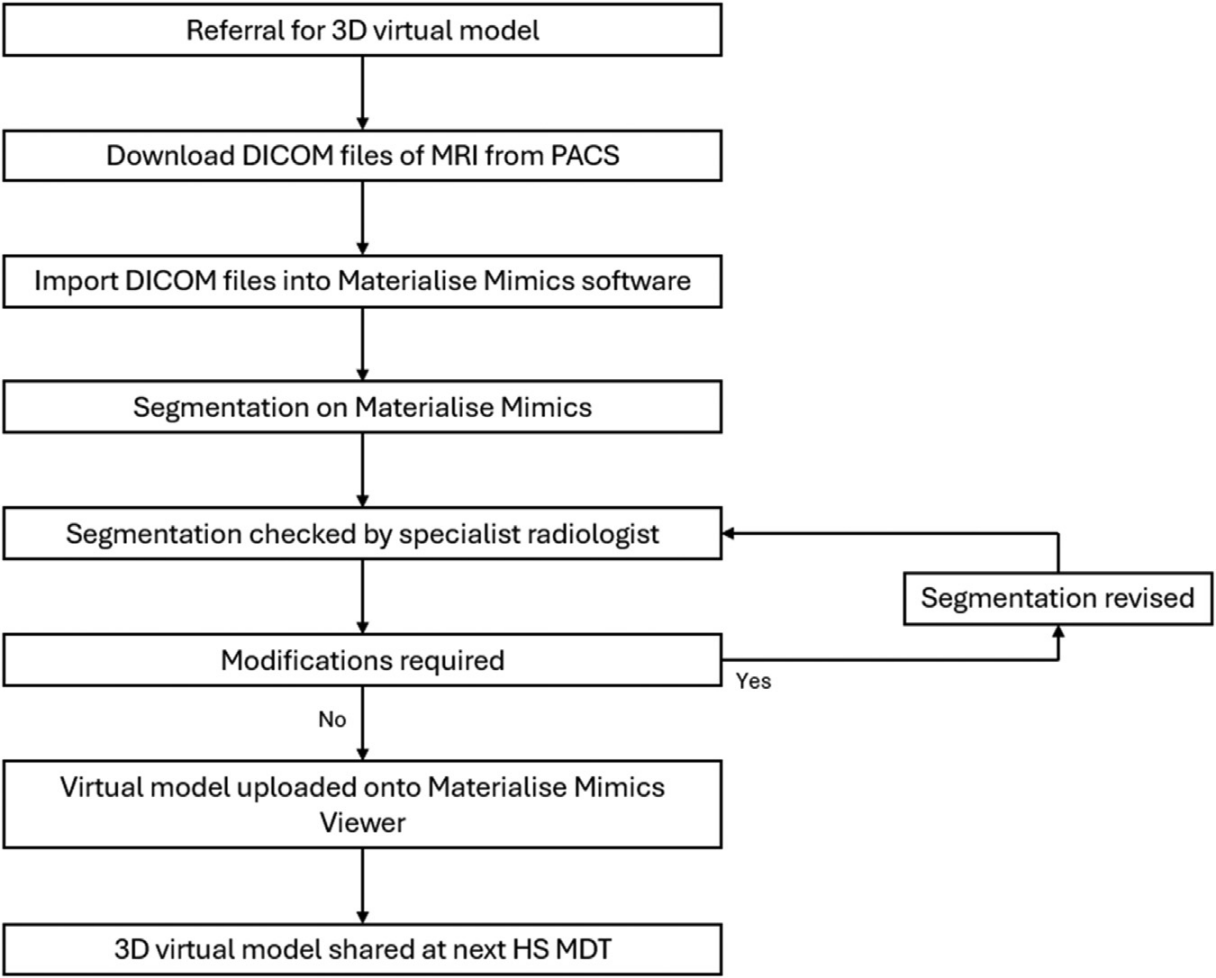
Workflow for 3D modeling.

The MRI sequences of the scan are then downloaded from PACS (picture archiving and communication system) in a DICOM format and imported into Materialize Mimics software. Existing licenses for the software at the Australian Centre for Complex Integrated Surgical Solutions (ACCISS) – which is based at our institution – are used for this purpose. Specific anatomical features segmented are HS disease and fistulous tracts, rectum, external anal sphincter, internal anal sphincter, puborectalis muscle, skin, and bony structures of the pelvis, femur and/or sacrum. Additional structures may be segmented depending on a case-by-case basis. These 3D virtual models are reviewed by a specialist radiologist prior to finalization and distribution. Screenshots of the 3D model from different views are taken and uploaded onto the patient’s electronic medical record (Figure 2). The virtual model is also uploaded onto Materialize Mimics Viewer, an online platform whereby 3D virtual models can be uploaded and viewed from any device, so that it is ready to be shared at the next MDT. At the following MDT that the patient is discussed, the virtual model can be used to supplement presentation of the structural imaging findings. We will also often use the virtual model for patient education to help them understand the extent of their disease and to help explain the operative plan relative to their anatomy.

In summary, patients with complex HS are discussed at our monthly MDT. Each case is discussed and after clinical assessment by the multidisciplinary team, a decision is made which involves either treatment or need for further investigations. At this point, the patient is referred for an MRI and/or 3D virtual modeling. Once they have completed their MRI, the 3D virtual model is created in preparation for re-discussion at the following MDT.

For the purposes of this case series, an existing dataset of eligible patients was accessed from a secure drive on Queensland Health computers according to the ethical application and study protocol. Inclusion criteria were patients with anogenital HS who had 3D virtual models created through MDT and who provided verbal consent for participation in this study. Data was separated at the patient level to ensure that images did not appear across training, validation and testing datasets.

## Case series

Patient 1 is a 42-year-old male with Hurley stage 3 HS with recurrent perianal and bilateral axilla disease. He previously had multiple surgeries under general surgery involving nine incision and drainages, examination under anesthesia and insertion of setons in the perianal region. Despite these interventions and being on infliximab (TNF inhibitor), he had progressive worsening of his perianal disease and the decision was made to create a defunctioning loop sigmoid colostomy. He was then referred to the PAH dermatology team due to the complexity of his case for MDT review. Clinically, he had constant draining and swelling of perianal abscesses with associated pain. MRI scan showed numerous subcutaneous ramifying fistulous tracts in the perianal region involving bilateral buttocks. There was also extensive involvement of the anal sphincteric complex with intersphincteric fistulous tracts and supralevator extension. A virtual model was requested by the MDT to help with surgical planning. The model helped to establish options for the patient, which involved either extensive surgery or less extensive deroofing surgery with aim to preserve sphincter function. In light of the surgical risk as demonstrated by the imaging and 3D model, the patient elected to proceed with the less extensive surgical option. The patient recovered well after his deroofing surgery. He has ongoing severe fistulous disease with expected residual sphincteric disease, confirmed on a recent examination under anesthetic by the colorectal team. He remains stable on Humira and is for regular follow-up by both the dermatology and colorectal surgery teams.

Patient 2 is a 29-year-old female with Hurley stage 2 HS of the buttocks and groin. She had suspected pre-diabetes and was only on topical therapy at the time of referral. Clinically, she had a draining sinus of her R buttock which was bothering her the most. She was discussed at HS MDT with an MRI showing a transsphincteric fistula tract which had localized active inflammation. A 3D model was created to help with surgical planning for deroofing surgery. The patient subsequently underwent deroofing as a combined colorectal and plastic surgery case. At the 18 month post-operative review, she was found to have completely healed her wounds with no recurrence.

Patient 3 is a 59-year-old male with severe long-standing Hurley stage 3 HS. On background, he has type-2 diabetes mellitus and is in remission for lung cancer treated with a right upper lobectomy. Management included cosentyx (generic name for drug – secukinumab) and multiple incision and drainages previously but no prior deroofing surgery. Clinically he was most bothered by his right lateral buttock disease as he was unable to sit due to pain. He was discussed at HS MDT and an MRI was performed to assess the extent of the disease. The MRI showed extensive disease affecting both buttocks, with greater involvement on the right, consistent with the clinical picture. The perineum and perianal regions were also affected with intersphincteric extension and multiple transsphincteric fistulas. Superiorly, the disease was also seen to extend to the level of the puborectalis muscle anteriorly. A 3D model was performed to better visualize the extent of the disease. The model helped the MDT make the final decision of recommending excision of the right lateral gluteal HS. The patient underwent deroofing surgery for the right gluteal disease. Despite healing well post-operatively, he unfortunately had recurrence of his right gluteal disease. He was discussed at the HS MDT again and there was a discussion between extensive surgery with a stoma given sphincteric involvement versus serial deroofing procedures of the symptomatic areas. The patient was not keen on a stoma and elected for the latter option. He has since had further deroofing of his right buttock disease.

Patient 4 is a 62-year-old male with Hurley stage 3 HS in the setting of Crohn’s disease on Humira (generic name of drug - adalimumab). He was discussed in HS MDT and the combination of the MRI findings and 3D model were used to help plan his deroofing surgery. The 3D model was able to clearly show the precise location of the intersphincteric tract with a visible seton-in-situ and continuation of the tract to the natal cleft. The patient has been booked and consented for deroofing surgery and is awaiting surgery.

Patient 5 is a 28-year-old male with Hurley stage 2 HS with L scrotal and R groin disease on Humira. He previously had natal cleft and R groin disease which resolved with deroofing. Three-dimensional modeling of his latest and previous MRI scans were requested at the HS MDT for teaching purposes as he was a case that responded well to medical treatment. He remains well with no concerns regarding his HS disease.

Patient 6 is a 27-year-old male with Hurley stage 3 HS on Humira (or adalimumab). He was seen at HS MDT and had multiple draining tracts of the L buttock which were clinically close to the anal sphincter. Decision was made to re-discuss patient 6 with MRI and a 3D model to better visualize the disease and its extent given proximity to anal sphincter. Fortunately, the disease did not involve the external anal opening and the patient was booked for deroofing surgery.

Patient 7 is a 52-year-old female with severe Hurley stage 3 HS. She had clinical anal sphincter involvement with severe perineal, perianal and vulva involvement. The plan from her initial HS MDT was for an MRI and 3D model. Imaging showed extensive fistulous disease extending from the external anal opening to the puborectalis with an intersphincteric collection and disease across bilateral buttocks near the anal opening. Upon re-discussion, consensus was that she would require extensive surgery and a diverting stoma to optimize post-operative healing of the deroofing site. The patient has received a diverting stoma and subsequent deroofing surgery with BTM application to the wound. She has had successful integration of her BTM and skin graft and has healed her wounds. She has residual smaller areas of disease which is being managed by dermatology in the outpatient setting Table 1.

**Table 1:** Clinical and 3D virtual images.

## Discussion

HS MDTs have become a crucial part of the treatment of complex HS patients at our institution due to the complex nature of our patient cohort. It is a unique construct within Queensland that offers a multidisciplinary approach to the management of complex cases that may have reached the limit of their options available locally.

Imaging has become an important tool in the accurate diagnosis of HS disease. Anogenital HS disease is not only one of the most common sites affected but also may be associated with the most debilitating consequences due to its proximity to the anal sphincter complex and other structures involved in fecal continence. MRI is particularly useful for imaging anogenital HS due to its ability to accurately capture deeper structures. Due to advances in 3D technology, 3D virtual reconstructions of structural imaging modalities can be offered to help visualization of complex structures for surgeons and patients.

With this case series, we are hoping to address gaps in the literature which include integration of 3D technology in routine clinical workflows and use of cross-sectional imaging in the management of cutaneous chronic inflammatory disorders. The literature outlines the value of imaging machine learning in improving the accuracy of diagnosing cutaneous conditions in dermatology. Shehu et al.^12^ and Karimi et al.^13^ discussed the importance of selecting important features for analysis to improve accuracy of computational analysis. Saba,^14^ Javet et al.^15^ and Nasir et al.^16^ applied this concept with clinical photography to improve accuracy of diagnosis of cutaneous dermatological conditions. Currently, the literature focuses on the development of classification systems and improvement in diagnostic accuracy for oncological purposes and/or use of 2-dimensional modalities. However, with this case series we hope to address gaps in the literature and expand this scope further to incorporate computerized training systems with structural imaging.

There are still a few limitations to the accessibility of these services. MRI scans can be time-consuming and time on the magnet is at a premium, meaning that there is often a delay in obtaining the correct MRI images. This means that scans may take months for patients to get their scans. During this time, there is the potential that there is a change in the patients’ symptoms or progression of the disease. We have had some success as a referral center by providing peripheral hospitals with our specific MRI protocol which allows patients to have their imaging closer to home whilst remaining under the care of the referral center for workup and segmentation in preparation for clinic evaluation and MDT discussion which is performed centrally. Furthermore, the manual segmentation of the anatomy and disease is labor intensive and subject to a learning curve for accuracy as there is still no established automated segmentation method for this indication. In addition, the sample size of this case series is limited and may not represent clinical diversity, limiting applicability of explainability analysis. It would be helpful in the future to generate a larger dataset and consider incorporating computerized training systems to aid diagnosis of complex conditions such as HS. Whilst the current literature is supportive of machine learning in aiding diagnosis of cutaneous malignancies using clinical images,^12^ we believe there is potential to expand this in the future with structural imaging.

In conclusion, we believe that there is value in the use of 3D virtual reconstructions for complex anogenital HS cases to aid clinicians in making decisions and help patients understand their disease. Hopefully with further optimizations in the MRI sequences, improvements in 3D modeling technology and better understanding of the nature of the disease, the quality and accessibility of these imaging modalities can be improved. Furthermore, we believe there is a potential to incorporate this workflow with developing artificial intelligence technologies in the management of complex anogenital HS and for educational purposes.

## CrediT authorship contributions

Conceptualization: Dr A.Y. Methodology: Dr A.Y. Data Curation: Dr A.Y. Formal Analysis: Dr A.Y. Investigation: Dr A.Y. Writing – Original Draft: Dr A.Y., Dr D.B., J.P. Writing – Review and Editing: all authors. Supervision: Dr T.L., Dr E.M.

## Funding

In-kind by Metro South Health.

## Ethical approval

Ethical approval was obtained with reference number HREC/2025/QMS/122018.

## Declaration of competing interest

There is no perceivable conflict of interest.
